# In Situ Forming Bioartificial Hydrogels with ROS Scavenging Capability Induced by Gallic Acid Release with Potential in Chronic Skin Wound Treatment

**DOI:** 10.3390/gels9090731

**Published:** 2023-09-09

**Authors:** Rossella Laurano, Alessandro Torchio, Gianluca Ciardelli, Monica Boffito

**Affiliations:** 1 Department of Mechanical and Aerospace Engineering, Politecnico di Torino, 10129 Torino, Italy; alessandro.torchio@polito.it (A.T.); gianluca.ciardelli@polito.it (G.C.); monica.boffito@polito.it (M.B.); 2 Department of Life Sciences, University of Modena and Reggio Emilia, 41125 Modena, Italy; 3 Institute for Chemical-Physical Processes, National Research Council (CNR-IPCF), 56124 Pisa, Italy

**Keywords:** bioartificial hydrogels, ROS scavenging, gallic acid, amphiphilic poly(ether urethane)s, drug delivery carriers, thermo-sensitive formulations, intracellular ROS measurements

## Abstract

In normal chronic wound healing pathways, the presence of strong and persistent inflammation states characterized by high Reactive Oxygen Species (ROS) concentrations is one of the major concerns hindering tissue regeneration. The administration of different ROS scavengers has been investigated over the years, but their effectiveness has been strongly limited by their short half-life caused by chronic wound environmental conditions. This work aimed at overcoming this criticism by formulating bioartificial hydrogels able to preserve the functionalities of the encapsulated scavenger (i.e., gallic acid—GA) and expand its therapeutic window. To this purpose, an amphiphilic poly(ether urethane) exposing -NH groups (4.5 × 10^20^ units/g_polymer_) was first synthesized and blended with a low molecular weight hyaluronic acid. The role exerted by the solvent on system gelation mechanism and swelling capability was first studied, evidencing superior thermo-responsiveness for formulations prepared in saline solution compared to double demineralized water (ddH_2_O). Nevertheless, drug-loaded hydrogels were prepared in ddH_2_O as the best compromise to preserve GA from degradation while retaining gelation potential. GA was released with a controlled and sustained profile up to 48 h and retained its scavenger capability against hydroxyl, superoxide and 1′-diphenyl-2-picrylhydrazyl radicals at each tested time point. Moreover, the same GA amounts were able to significantly reduce intracellular ROS concentration upon oxidative stress induction. Lastly, the system was highly cytocompatible according to ISO regulation and GA-enriched extracts did not induce NIH-3T3 morphology changes.

## 1. Introduction

Reactive Oxygen Species (ROS) are radical or nonradical oxidizing agents commonly produced by monocytes and neutrophils immediately after injury, i.e., during the first phase of the wound closure pathway [1]. Indeed, they play a crucial role in protecting the wound environment from external pathogens [2] and promoting tissue healing by enhancing angiogenesis [3], and by working as mediators in intracellular signaling [4]. However, due to their extreme reactivity, high concentrations of ROS resulting from excessive production or insufficient detoxification can lead to the achievement of a persistent oxidative stress state with deleterious consequences. For instance, the literature reports that ROS accumulation in the wound site not only results in chronic wound inflammation, but also hinders tissue healing and enhances cell transformation towards neoplastic phenotypes [5,6]. Therefore, the administration of ROS scavengers is a promising strategy in the treatment of chronic skin wounds aiming at reducing oxidative stress and enhancing wound closure.

However, irrespective of the adopted ROS scavenger administration route (i.e., systemic or topical), specific attention should be paid to minimizing side effects and maximizing their benefits. Indeed, systemic administration of ROS scavengers could result in their nonspecific diffusion thus, potentially altering the metabolism of non-target tissues. Furthermore, due to the quick renal clearance, high doses and multiple administrations/day are required to ensure the achievement of an effective therapeutic window. On the other hand, the topical administration of ROS scavengers could result in their easy loss of function because of degradation phenomena enhanced by the chronic wound hostile environment (e.g., strong alkalinity of wound exudate and presence of microbial species). 

To overcome these critical issues, several types of ROS scavenger delivery systems have been engineered, such as liposomes and organic and inorganic nanoparticles [7,8,9]. Among them, hydrogels can be considered a powerful delivery platform providing several beneficial features in wound treatment. Indeed, such systems can effectively and simultaneously combine their attractive potentialities as delivery carriers and wound dressings, being able to fulfill the requirements identified by the Moist Wound Healing theory [10]. Specifically, hydrogel-based delivery carriers can provide: (i) ROS scavenger protection from degradation phenomena by entrapping such molecules in their network; (ii) mini-invasive administration through injection in the wound cavity; (iii) proper environmental conditions (e.g., constant temperature, humidity, fluid absorbing capacity) towards tissue healing, and (iv) easy shape adaption to the wound cavity with no pressure over perilesional tissues.

Among investigated ROS scavengers (e.g., glutathione, lipoic acid, vitamins C and E), gallic acid (GA) has been selected in this work as a naturally derived therapeutic agent for the treatment of hard-to-heal wounds due to several beneficial pharmacokinetics properties [11]. Such polyphenolic compound is found in a wide variety of vegetables, plants, fruits, coffee, tea, and wine in the form of free acids, esters or hydrolyzable tannins [12]. From a pharmacological point of view, GA not only exerts antioxidant properties but is also known to show antibacterial [13], antitumorigenic [14] and anti-inflammatory features [15]. For these reasons, it has found application for the treatment of many disorders, such as prostate [16] or tobacco-related [17] cancers, as an antioxidation promoter in wound healing [18] or as an anti-inflammatory drug in the prevention of inflammation-induced myometrial contractions and premature rupture of fetal membranes [19]. However, due to its labile stability in physiological-like fluids, particular attention must be paid to the design of properly engineered GA delivery carriers which should be able to preserve its pharmacological features during administration [12]. For instance, recently published works report the encapsulation of GA into purely lipid-based [20,21,22] and polymer-based nanocarriers [23,24,25] or in lipid–polymer hybrid systems [26,27,28] showing improved biopharmaceutical properties compared to the direct release of GA by itself. Based on these premises, in this work GA was not used as such, but it was embedded in a hydrogel-based delivery carrier with the aim to prolong its half-life and thus its therapeutic effects.

More in detail, this work aimed at developing a thermosensitive bioartificial macromolecular hydrogel as a protective carrier for the topical delivery of GA in the wound bed through mini-invasive injection. Hydrogel polymeric components were selected to show bioactive properties and to exert proper functions. Specifically, an amphiphilic poly(ether urethane) (PEU) exposing secondary amino groups was first synthesized to ensure hydrogel thermo-sensitivity and to exert antimicrobial and antifungal properties [29]. On the other hand, low molecular weight hyaluronic acid (HA) was selected based on its renowned pro-angiogenic and tissue regeneration features [30,31,32,33,34]. The bioartificial hydrogel thermo-sensitivity as well as their responsiveness to physiological-mimicking fluids were studied through rheological and in vitro swelling tests, respectively. Then, GA-loaded hydrogels as delivery carriers were investigated through in vitro drug release tests. Lastly, the capability of released GA to work as a ROS scavenger as well as its cytocompatibility were assessed through in vitro scavenging assays and by measuring the intracellular ROS concentration upon treatment and through immunofluorescence staining, respectively. 

## 2. Results and Discussion

One of the major concerns hindering the proceeding of the normal healing pathway is the presence of a persistent and prolonged excessive ROS microenvironment finally leading to the achievement of a chronic wound state [35]. Indeed, high ROS concentrations strongly inhibit the activity of physiologically produced antioxidant enzymes responsible for the control over the inflammation state and the regulation of oxidative stress levels [36]. Hence, the administration of ROS scavenging molecules is a promising strategy to reduce the in situ ROS concentration and promote the achievement of the following phase towards tissue healing. In this context, the naturally derived GA was selected as a ROS scavenger in this work. To overcome issues related to its short half-life when released in a strong oxidative environment, an injectable bioartificial hydrogel was engineered as a GA delivery carrier with the aim of protecting the molecule and prolonging its therapeutic window. Specifically, hyaluronic acid was selected as a hydrogel natural component due to its renowned pro-angiogenic and pro-regenerative properties [37,38], while an amphiphilic poly(ether urethane) was ad-hoc synthesized in order to be able to interact with HA polymeric chains, thus resulting in an in situ forming hydrogel. More in detail, the PEU here-exploited resulted from a previously optimized synthesis procedure [29]. Hence, only the main PEU chemical properties were briefly reported in this work, while for an in-depth discussion, the reader could refer to Laurano et al. [29].

### 2.1. Poly(Ether Urethane) Chemical Characterization

**Figure 1:**
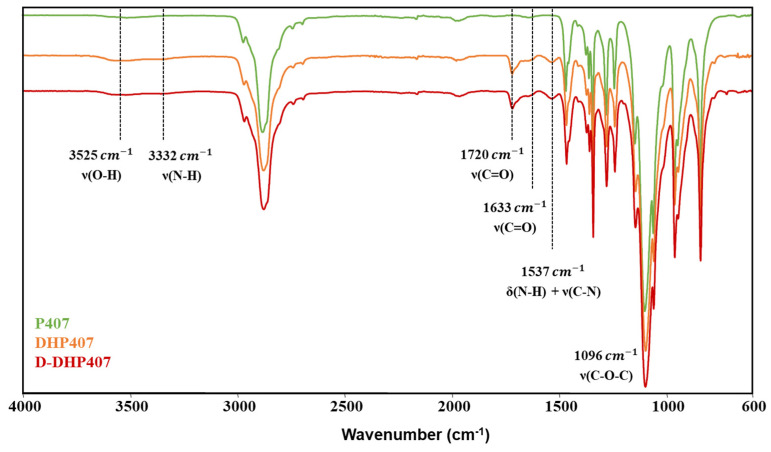
Attenuated Total Reflectance Fourier Transform Infrared (ATR-FTIR) spectra of Poloxamer^®^ 407 (green), DHP407 (orange) and D-DHP407 (red). Highlighted bands proved the successful PEU synthesis and the preservation of urethane bond integrity as a consequence of Boc removal.

**Figure 2:**
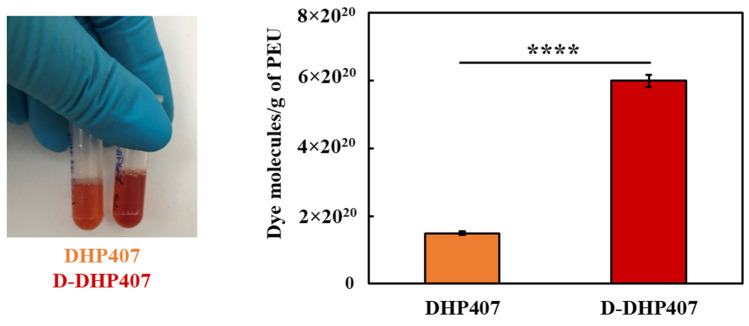
Representative image of DHP407 and D-DHP407 samples subjected to Orange II Sodium Salt assay (**left**) and quantification of dye molecules detected per g of polymer (**right**). The number of free amino groups resulted from the difference between dye molecules measured for D-DHP407 and DHP407 samples. Statistical significance: **** = *p* < 0.0001.

The successful synthesis of a high molecular weight PEU bearing secondary amino groups along the polymeric chains (i.e., D-DHP407) was step-by-step assessed through Attenuated Total Reflectance Fourier Transform Infrared (ATR-FTIR) spectroscopy (Figure 1) and Size Exclusion Chromatography (SEC). Moreover, the colorimetric Orange II Sodium Salt assay was exploited to quantify the secondary amines exposed as a consequence of Boc group removal through an acid treatment (Figure 2). Specifically, the comparison between Poloxamer^®^ 407 (P407) and Boc-protected PEU (i.e., DHP407) ATR-FTIR spectra showed the characteristic peaks proper of the macrodiol (e.g., the C-O-C stretching vibration at 1096 cm^−1^) and the appearance of new vibrational bands ascribed to the formation of urethane bonds. Specifically, the N-H stretching and bending vibrations at 3332 cm^−1^ and 1537 cm^−1^, respectively, the C=O stretching at 1720 cm^−1^ and 1633 cm^−1^, and the stretching of the C-N bonds at 1537 cm^−1^ [39]. Moreover, no differences were detected between DHP407 and D-DHP407 spectra, thus suggesting the absence of chemical bond degradation induced by the acidic removal of Boc caging groups. These results were further supported by SEC analyses giving ${\bar{M}}_{n}$ and D values equal to 9 kDa and 1.3, 20 kDa and 1.6 and 20 kDa and 1.6 for P407, DHP407 and D-DHP407, respectively, according to our previous findings [29].

Moreover, the colorimetric Orange II Sodium Salt assay proved the exposure of secondary amines. Indeed, although both DHP407 and D-DHP407 gave orange solutions when in contact with the dye [40], the absorbance intensity measured for D-DHP407 samples was significantly higher compared to the control, resulting in 4.5 × 10^20^ secondary amino groups/g of polymer.

### 2.2. Bioartificial Hydrogel Properties in Physiological-Mimicking Conditions

Bioartificial HA/D-DHP407 hydrogels were prepared at different overall concentrations ranging between 10% and 15% *w*/*v* and by using double demineralized water (ddH_2_O) or Dulbecco’s Phosphate Buffered Solution (DPBS) as solvents (Table 1). Irrespective of the tested composition, HA and D-DHP407 were blended at 50/50 weight ratio. Polymer miscibility and the absence of phase separation phenomena in quiescent conditions and at different temperatures (i.e., 4 °C and 37 °C) were previously investigated at the macro-, micro- and nano-scale, and findings were reported in a recently published work [41]. In this work, we first investigated the influence of dissolved ions in the solvent used for polymer dissolution over gelation mechanism and kinetics through the qualitative tube inverting test. Then, the most promising HA/D-DHP407 formulations in terms of polymeric concentration were further studied through rheological tests and in vitro hydrogel responsiveness to physiological-mimicking fluids.

#### 2.2.1. Hydrogel Thermo-Responsiveness

A noteworthy thermo-responsive behavior was observed for DPBS-based systems at 37 °C. In fact, irrespective of hydrogel polymeric concentration, all DPBS-based bioartificial formulations showed a gelation time of 3 ± 1 min. As a comparison, pure D-DHP407-based solutions at concentrations of 10%, 12% and 15% *w*/*v* required an incubation time equal to 12.3 ± 0.6, 9.3 ± 0.6 and 8.3 ± 0.6 min, respectively, to undergo gelation [29]. This observation suggested the occurrence of constructive interactions between HA and D-DHP407 at different levels, thus resulting in enhanced gelation kinetics. Specifically, the temperature-induced hydrogel network could be ascribed toa particular configuration resulting from the combination of “supramolecular” and “macromolecular” arrangements [42]. In fact, poly(ether urethane)s based on P407 macrodiol (like D-DHP407) show the ability to self-organize into micelles upon temperature increase as supramolecular structures [40,43,44]. These ordered structures can further interact with linear macromolecules, such as HA, thus producing a network having both “supramolecular” and “macromolecular” facets. This hypothesis was further corroborated by Dynamic Light Scattering analyses previously performed on formulations based on HA and a similar PEU exposing thiol groups [41]. Specifically, such nano-scale investigation demonstrated that HA polymeric chains did not interfere with PEU’s capability to organize into micelles; moreover, at 37 °C strong interactions occurred between thiol groups and HA chains which probably formed a hydrated shell around them [40]. As both -NH and -SH groups are hydrophilic moieties, it is reasonable to suppose that they behave similarly upon polymer solubilization in an aqueous medium. Moreover, the presence of -COOH and -NH groups along HA and D-DHP407 polymeric chains, respectively, further enhanced the occurrence of electrostatic interactions among them. Despite these strong polymeric interactions, the gelation process was totally reversible: the hydrogels showed a gel-to-sol transition when cooled to low temperature (i.e., 3 °C). Nonetheless, such peculiarities were restricted to the presence of dissolved ions within saline solutions. Indeed, the presence of salts in a watery environment containing polymers in solution can induce a salting-out effect, which is generally responsible for enhancing the formation of hydrophobic interactions and homogeneous precipitation of polymeric complexes [45,46,47]. Moreover, the occurrence of hydrogel network development was simultaneous to an evident turbidity increase.

**Figure 3:**
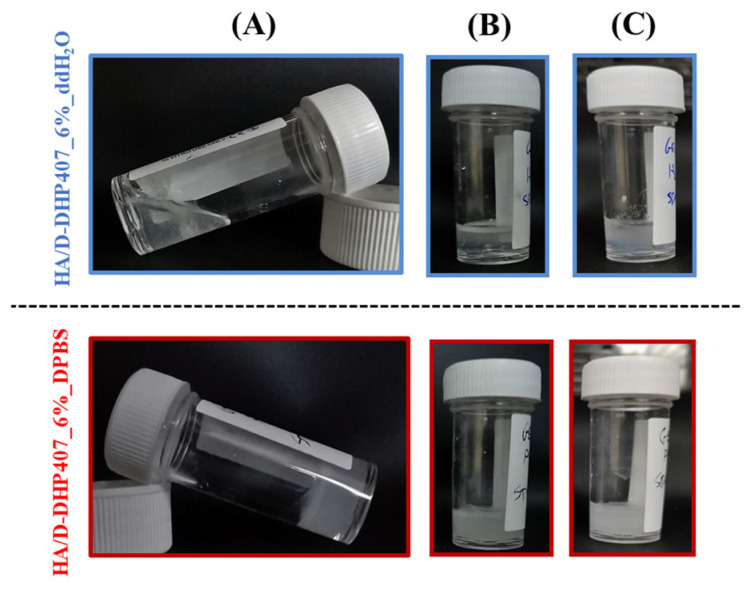
Representative images of HA/D-DHP407 formulations prepared by using ddH_2_O (upper) and DPBS (lower) as solvents and their corresponding behavior at physiological temperature with or without contact with a saline solution. (**A**) System equilibration at 37 °C for 5 min; (**B**) system equilibration at 37 °C for 5 min followed by DPBS contact for 30 min; (**C**) system equilibration at 37 °C for 5 min followed by DPBS contact for 2 h.

On the other hand, HA/D-DHP407 samples prepared in double demineralized water did not show a sol-to-gel transition upon temperature increase and maintained a transparent appearance, thus indicating the absence of relevant and homogeneous precipitation phenomena as factors for hydrogel network formation upon thermal stimulus. However, the solutions composed of pure water retained the possibility to undergo a sol-to-gel transition when in contact with saline environments, such as wound exudates [48]. Indeed, while HA/D-DHP407_ddH_2_O formulations remained in the sol state at 37 °C irrespective of incubation time, their contact with DPBS progressively induced a sol-to-gel transition as visually observed through the propagation of turbidity across the entire hydrogel thickness (Figure 3). Even though preliminary, these results suggested a notable tunability of the physical properties characterizing the here-investigated hydrogels, thus widening the range of possible applications in the field of chronic wounds.

**Figure 4:**
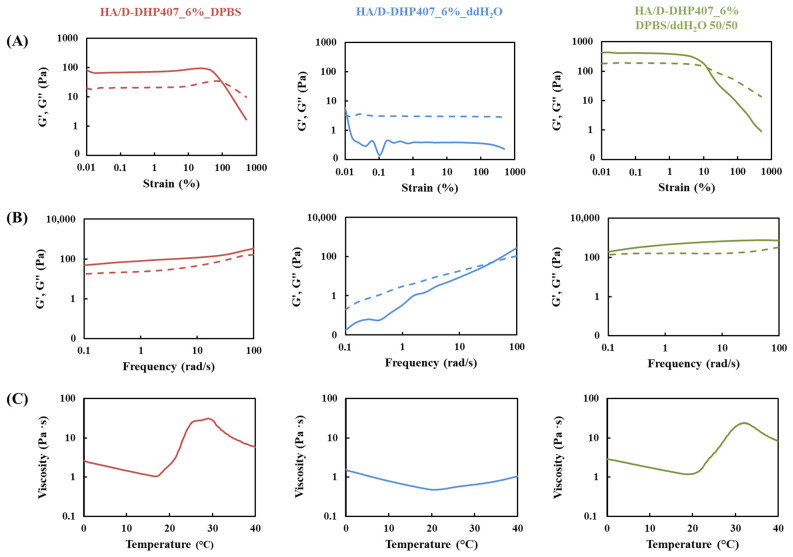
Rheological characterization of HA/D-DHP407_6% formulations prepared by using pure DPBS (red), pure ddH_2_O (blue), and DPBS/ddH_2_O 50/50 *v*/*v* (green) as solvents. Continuous and dashed lines represent the storage (G′) and loss (G″) moduli, respectively. (**A**) Strain sweep test performed at 37 °C by applying 0.01–500% strain (frequency 1 s^−1^). (**B**) Frequency sweep test at 37 °C within 0.1 and 100 rad/s (0.1% strain). (**C**) Viscosity profile acquired through temperature ramp test within 0–40 °C, 1 °C/step (frequency 10 s^−1^). Graphs in panels A and B show a Log scale for both X-axis and Y-axis, while graphs in panel C show a Log scale for Y-axis and a decimal scale for X-axis.

The consistency of these qualitative findings was further investigated through rheological tests. To this purpose, the formulation containing HA at 6% *w*/*v* and D-DHP407 at 6% *w*/*v* (i.e., HA/D-DHP407_6%) was selected, as it represented an intermediate condition with promising features in terms of mixability, processability and stability. More in detail, besides HA/D-DHP407_6%_ddH_2_O and HA/D-DHP407_6%_DPBS formulations, an intermediate condition using DPBS/ddH_2_O at 50/50 *v*/*v* as solvent (i.e., HA/D-DHP407_6%_DPBS/ddH_2_O) was considered, aiming at further studying the gelation mechanism occurring upon the absorption of external saline fluids (Figure 4).

As expected, both strain sweep and frequency sweep tests conducted at 37 °C confirmed the qualitative observations from the tube inverting test. Indeed, while a gel state was achieved by HA/D-DHP407_6%_DPBS with storage modulus (G′) values higher than loss modulus (G″) ones, the maintenance of a sol state was observed for the same formulation prepared by using pure ddH_2_O (i.e., G′ < G″). Moreover, results from the hybrid formulation (i.e., HA/D-DHP407_6%_DPBS/ddH_2_O 50/50) further supported our previous hypothesis on the salt-induced gelation mechanism [49,50]. Indeed, G′ values higher than G″ ones were registered for HA/D-DHP407_6%_DPBS/ddH_2_O 50/50 representative of a gel state at 37 °C, as in the case of HA/D-DHP407_6%_DPBS, but a higher G′-G″ difference at 0.01% strain (i.e., approx. 239 Pa vs. 61 Pa) and a lower strain at break (i.e., 3.5% vs. 90%) were observed compared to HA/D-DHP407_6%_DPBS, thus suggesting the achievement of a stronger gel network. However, despite both systems were able to macroscopically keep their shape, they could not be defined as completely developed hydrogels from a rheological point of view [51].

Acquired viscosity profiles as a function of temperature increase were in complete accordance with previous observations: while a sharp viscosity increase was registered for both HA/D-DHP407_6%_DPBS and HA/D-DHP407_6%_DPBS/ddH_2_O 50/50 systems upon heating, no remarkable changes in viscosity values were observed for HA/D-DHP407_6%_ddH_2_O formulations within the entire temperature range, meaning that temperature increase was not able to induce gelation. Moreover, the comparison between pure DPBS and hybrid formulations further evidenced the role exerted by dissolved ions in the system to induce and enhance the transition from the sol to the gel state. Indeed, the temperature marking the beginning of viscosity increase was measured to be 17.4 °C and 19.7 °C for HA/D-DHP407_6%_DPBS and HA/D-DHP407_6%_DPBS/ddH_2_O 50/50, respectively, suggesting that the former system required lower temperature to start the polymeric chain arrangement process due to the presence of a higher salt content. However, this faster chain aggregation in HA/D-DHP407_6%_DPBS resulted in the imminent formation of bigger aggregates which, in turn, slightly hindered the achievement of a well-structured gel network compared to HA/D-DHP407_6%_DPBS/ddH_2_O 50/50.

#### 2.2.2. Hydrogel Responsiveness to Physiological-like Fluids

The ability of HA/D-DHP407-based hydrogel networks to respond to the external environment is an important feature in assessing their suitability as delivery systems. In fact, a proper delivery platform should release its payload without destabilizing the constituent network [51,52]. In that regard, the evaluation of hydrogel wet weight changes during incubation in contact with a physiological-like solution (i.e., DPBS) could be helpful.

**Figure 5:**
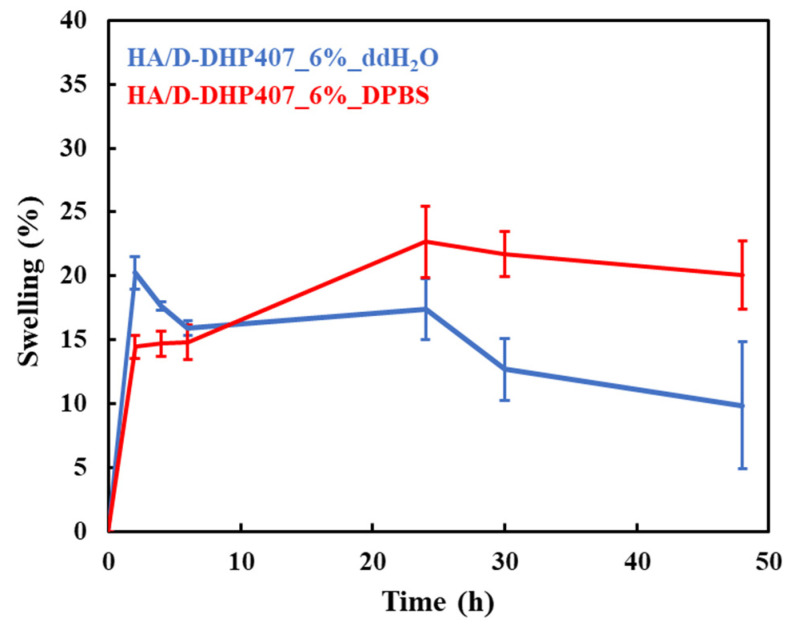
Swelling percentage profiles registered for HA/D-DHP407_6%_ddH_2_O (blue) and HA/D-DHP407_6%_DPBS (red) hydrogels in contact with DPBS up to 48 h at 37 °C. Statistical significance: 2 h = **; 4 h = **; 6 h = ns; 24 h = *; 30 h = **; 48 h = *. Legend: ** = 0.001 < *p* < 0.01, * = 0.01 < *p* < 0.05, ns = not significant.

As reported in Figure 5, all the hydrogels showed a relevant ability to absorb external fluids (i.e., around 15% swelling after only 2 h of incubation), whether the systems were based on DPBS or ddH_2_O.

The samples based on DPBS showed a slower increase in their wet weight with respect to the solutions composed of pure water. In this latter case, a noteworthy sol-to-gel transition was observed over incubation in contact with DPBS at 37 °C, in accordance with previously discussed findings. This peculiarity indicated that HA/D-DHP407_6%_ddH_2_O solutions were able to crosslink through the diffusion of ions from the external environment. Hence, they can be considered as in situ gelling bioartificial networks. In fact, their behavior was not consistent with the one characterizing unstable solutions, as they maintained their wet weight over time in a condition of positive swelling (i.e., without showing significant solubilization phenomena). Over time, HA/D-DHP407_6%_DPBS formulations showed a more sustained incremental fluid absorption, and then, their swelling percentage stabilized at around 20%. Generally, the behavior of the here-developed hydrogels can be considered promising for drug delivery applications in the field of chronic wounds. In fact, the high stability and fast responsiveness characterizing these networks represented valuable features for an effective release of therapeutic agents and additionally for the wound exudate wash-out through absorption [53].

### 2.3. In Vitro GA Release Test and Assessment of Its Scavenging Properties

**Figure 6:**
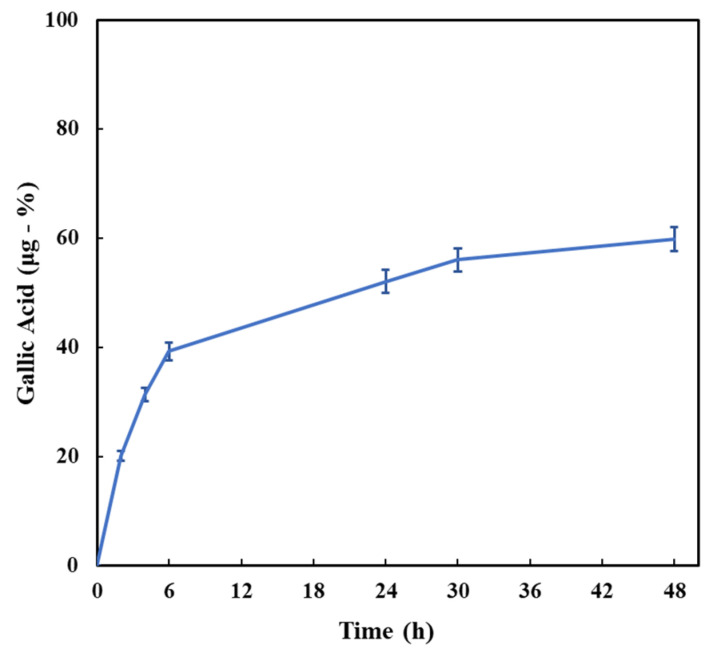
Gallic acid release profile obtained by incubating HA/D-DHP407_6%_ddH_2_O hydrogels in contact with DPBS up to 48 h at 37 °C. Released percentages correspond to released amounts of GA in µg. Statistical analysis was performed to study the progressive release of GA, i.e., by comparing the amounts released at each time point with the corresponding previous one. Statistical significance: 2 h = ****; 4 h = ****; 6 h = ****; 24 h = ****; 30 h = *; 48 h = *. Legend: **** = *p* < 0.0001, * = 0.01 < *p* < 0.05.

Based on their responsiveness to physiological-mimicking features, both HA/D-DHP407_6%_DPBS and HA/D-DHP407_6%_ddH_2_O hydrogels showed high potentialities as drug delivery carriers. However, due to the high instability of the selected therapeutic agent (i.e., gallic acid) for the here-presented application, superior attention should be paid to system engineering with the aim of maximizing the preservation of GA properties and the overall outcomes. For this reason, hydrogels based on pure water were selected. In fact, HA/D-DHP407_6%_ddH_2_O hydrogels were characterized by higher suitability for GA encapsulation, reducing the occurrence of premature oxidation enhanced by the presence of soluble salts [54]. Moreover, they showed the peculiar capability to undergo in situ gelation by absorbing external fluids, while maintaining a thermo-reversible behavior. As shown in Figure 6, GA was progressively released from the hydrogels with sustained kinetics. Indeed, no burst release was observed and the rate of GA delivery decreased over time, thus highlighting the progressive achievement of a plateau in the release profile. In addition, the rate of GA release during the entire duration of the test maintained drug concentration within the therapeutic window identified in the literature to treat the inflammatory state of wounds [55,56] and exert inhibitory and bactericidal effects [57].

The delivered amount of GA at 48 h of incubation resulted to be 59.8 ± 2.2 µg (i.e., 59.8 ± 2.2% of the total loaded GA within the hydrogels), thus suggesting a proper responsiveness of the systems, in accordance with previous findings from incubation tests in contact with a physiological-like environment. The combination of all results indicated the overall appropriateness of the engineered bioartificial hydrogels for wound treatment: they were able to absorb external fluids (e.g., exudates) while preserving their integrity and effectively releasing the encapsulated therapeutic agent without showing burst release. As a further demonstration, the Peppas release exponent was calculated according to Equation (2) and resulted to be 0.61 ± 0.01, thus indicating an intermediate mechanism of release between diffusion (exponent equal to 0.45) and swelling (exponent equal to 0.89). Hence, this model suggested a release process consisting of an anomalous transport, which could be considered optimal as it indicated an enhancement of drug release in addition to simple diffusion due to a notable process of mass exchange [44]. In that regard, the fractions of released HA and D-DHP407 could be generally helpful for wound healing [29,58,59] and drug encapsulation/delivery [51,60], respectively.

**Figure 7:**
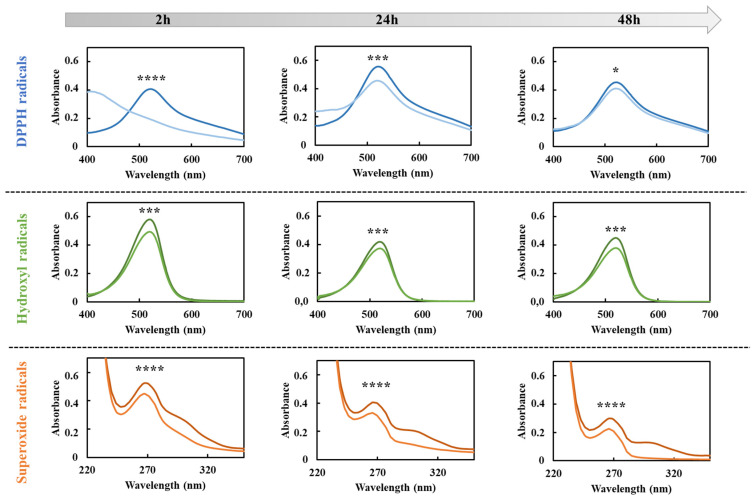
UV/Vis profiles exploited to quantify the scavenging activity exerted by released gallic acid at 2 h, 24 h and 48 h of incubation against DPPH (blue), hydroxyl (green) and superoxide (orange) radicals. Dark colors refer to control samples (i.e., samples not containing GA), while light colors stand for GA-loaded hydrogel extracts collected at each time point. Statistical significance assessed by comparing the absorbance values measured from GA-loaded and unloaded systems. Legend: **** = *p* < 0.0001, *** = 0.0001 < *p* < 0.001, * = 0.01 < *p* < 0.05.

To evaluate the preservation of released GA antioxidant properties, ROS scavenging activities against hydroxyl, 1′-diphenyl-2-picrylhydrazyl (DPPH) and superoxide radicals were in vitro assessed through the Fenton reaction and the DPPH and pyrogallol assays, respectively. Specifically, such assays were performed on GA released in DPBS from HA/D-DHP407_6%_ddH_2_O at different time points (i.e., 2 h, 24 h and 48 h). All these colorimetric tests were based on the evaluation of absorbance intensity changes resulting from GA interactions with radicals [61,62]. Figure 7 reports the comparison between the UV/Vis absorbance profiles acquired at each time point for GA-loaded HA/D-DHP407_6%_ddH_2_O extracts and their corresponding control condition (i.e., absence of gallic acid).

**Figure 8:**
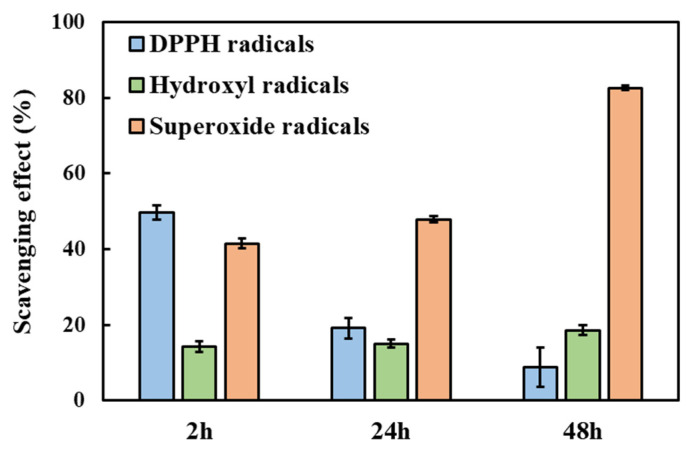
ROS scavenging activities exerted by GA released from HA/D-DHP407_6%_ddH_2_O hydrogels after 2 h, 24 h and 48 h of contact with DPBS at 37 °C. GA antioxidant features were in vitro tested against DPPH (blue), hydroxyl (green) and superoxide (orange) radicals. The scavenging trend was registered to be different over time based on the type of radical: steady, increasing and decreasing effects within 48 h of incubation for hydroxyl, superoxide and DPPH radicals, respectively.

Irrespective of the considered assay and time point, the released GA from HA/D-DHP407_6%_ddH_2_O hydrogels seemed to effectively exert scavenging activities through a decrease in the peak absorbance intensity compared to the control (i.e., at approx. 520 nm for DPPH and hydroxyl radicals and 299 nm for superoxide radicals). Moreover, these reductions were statistically significant irrespective of the considered condition. Specifically, such a feature can be attributed to GA molecules’ capability to act as electron donors and bind radicals present in the oxidative environment [63]. A quantitative evaluation of this scavenging activity was obtained by expressing the observed differences in absorbance intensity in terms of percentages with respect to their corresponding control condition (Figure 8). Irrespective of the considered reactive species, results evidenced the capability of released GA to effectively exert antioxidant properties over time up to 48 h, thus suggesting the chemical preservation of the molecule provided by the hydrogel up to its release. More in detail, a not so high but constant scavenging effect (approx. 15–20%) was registered against hydroxyl radicals, while higher values were obtained against DPPH and superoxide radicals. Moreover, the former showed a decreasing trend over time, thus suggesting a progressively reducing antioxidant capability of GA against DPPH; conversely, an opposite situation was observed for the latter with scavenging percentages against superoxide radicals equal to 82.6% ± 0.6% at 48 h of incubation.

### 2.4. In Vitro Evaluation of System Cytocompatibility

**Figure 9:**
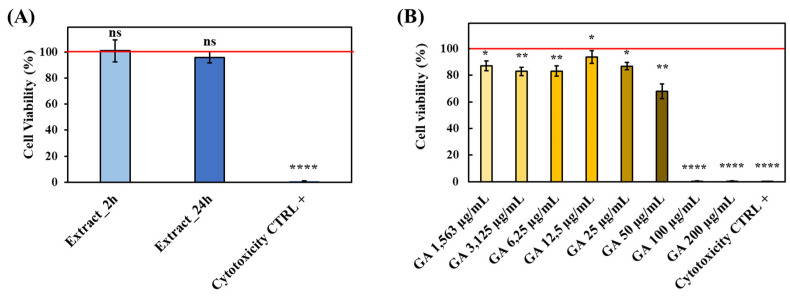
In vitro evaluation of system cytocompatibility according to the ISO 10993:5 regulation [64]. (**A**) Cytocompatibility tested on HA/D-DHP407_6%_ddH_2_O hydrogel extracts collected at 2 h and 24 h. (**B**) Dose/response curve of GA at concentrations ranging between 0 and 200 µg/mL. For both tests, cell viability percentages were calculated with respect to cells cultured in GA-free complete medium and represented by the horizontal red line. Statistical analysis performed by comparing each condition with the negative cytotoxicity control (i.e., cells cultured in complete medium). Legend: **** = *p* < 0.0001, ** = 0.001 < *p* < 0.01, * = 0.01 < *p* < 0.05, ns = not significant.

In view of hydrogel exploitation as a drug delivery carrier in the treatment of chronic skin wounds, the cytocompatibility of the hydrogel itself and the released GA was first assessed through cytocompatibility assays on murine fibroblasts conducted in accordance with the ISO 10993:5 regulation [64]. Results evidenced that the metabolic activity of murine fibroblasts cultured with hydrogel extracts (i.e., culture medium collected after 2 h and 24 h of contact with the hydrogels) was comparable to that of cells cultured in complete medium (Figure 9A).

On the other hand, the dose/response curve performed by varying GA concentration within 0–200 µg/mL revealed that all tested conditions up to 50 µg/mL (i.e., concentration higher than those registered in the in vitro drug release study) turned out to be cytocompatible as defined by ISO norms (i.e., cell viability higher than 70%, [64]) (Figure 9B).

Hence, these preliminary results confirmed the suitability of the engineered hydrogels to be exploited as GA delivery carrier avoiding the occurrence of any cytotoxic effects induced either by the polymeric component or by the encapsulated therapeutic agent.

### 2.5. GA Protective Effects under Induced Oxidative Stress

**Figure 10:**
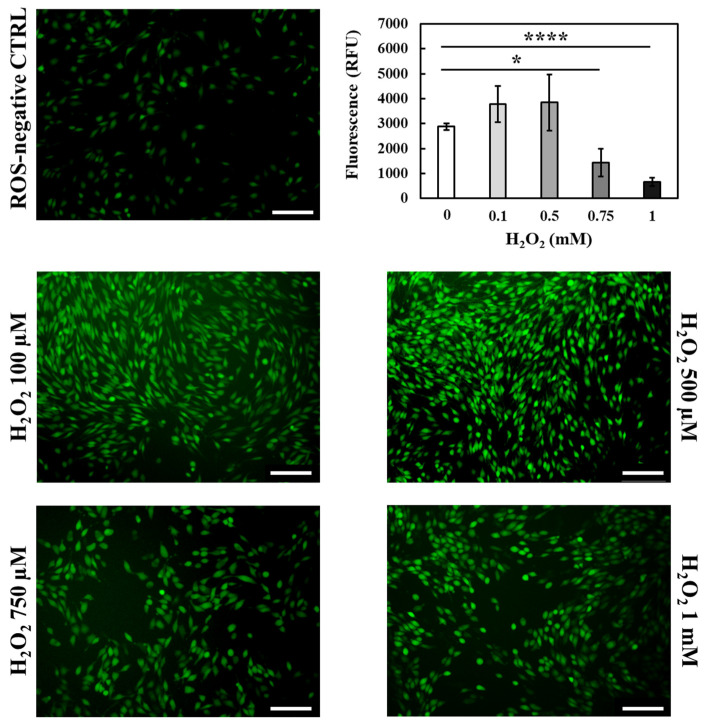
Qualitative and quantitative evaluation of NIH-3T3 oxidative stress induced by treating the cells with different concentrations of H_2_O_2_ ranging between 100 µM and 1 mM. Cells cultured in complete medium without the addition of H_2_O_2_ were used as ROS-negative control. Scale bar: 75 µm. Legend: **** = *p* < 0.0001, * = 0.01 < *p* < 0.05.

The presence of a strong oxidative environment resulting from ROS overproduction is known to exert deleterious effects on the surrounding cells by promoting DNA, lipid and protein damage [61]. In the case of chronic skin wounds such condition is the main cause hindering tissue healing and thus, wound closure [65]. Hence, a crucial point to overcome wound chronicity is to quickly reduce the in situ ROS concentration to levels able to promote the beginning of the repair process. Indeed, although ROS concentrations in vivo are difficult to determine due to their short half-life, studies revealed that 100–250 µM hydrogen peroxide (H_2_O_2_) concentrations are normally present at the wound site and considered safe for tissue healing [3]. Conversely, higher values could potentially be found in a persistent inflammation state. Hence, to evaluate the capability of GA released from HA/D-DHP407_6%_ddH_2_O hydrogels up to 48 h to effectively reduce the production of intracellular ROS, an in vitro oxidative environment was first induced. To this purpose, NIH-3T3 murine fibroblasts were first treated with different H_2_O_2_ concentrations to identify the most promising one able to induce oxidative stress without inducing cell cytotoxicity and death (Figure 10).

**Figure 11:**
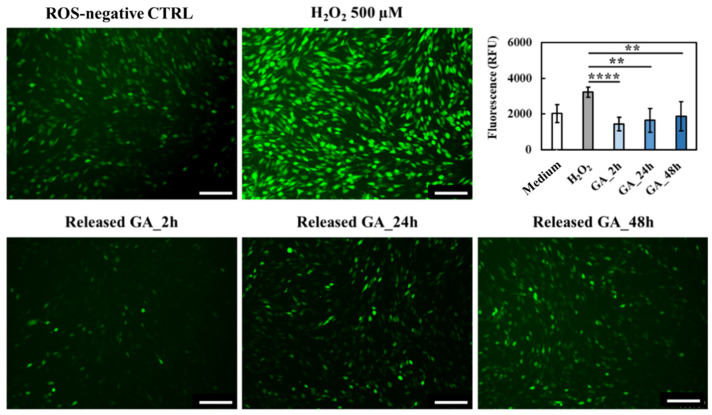
Qualitative and quantitative evaluation of released GA capability to reduce the concentration of intracellular ROS species in the presence of oxidative stress induced by treating NIH-3T3 murine fibroblasts with 500 µM H_2_O_2_. Scale bar: 100 µm. Statistical significance: **** = *p* < 0.0001, ** = 0.001 < *p* < 0.01.

Then, the successful production of intracellular ROS was assessed by using 2′,7′-dichlorodihydrofluorescein diacetate (DCFH-DA) staining. Indeed, DCFH-DA deacetylation by intracellular esterases followed by oxidation by intracellular ROS results in a highly fluorescent compound that can be easily detected and quantified [61]. Compared to the control, a strong increase in emitted fluorescence was observed up to 500 µM H_2_O_2_; conversely, further H_2_O_2_ concentration increase resulted in fluorescence values significantly lower with respect to the control. These observations could be attributed to H_2_O_2_-induced cytotoxicity which strongly reduced cell viability and, thus, DCFH-DA interaction with intracellular esterases [61]. Hence, 500 µM H_2_O_2_ was selected as a proper concentration able to induce oxidative stress while preserving cell viability and, thus, used to study the effects exerted by released GA. More in detail, in the presence of oxidative stress, cells were treated with GA-enriched extracts collected after 2 h, 24 h and 48 h of incubation, and intracellular ROS concentrations were measured (Figure 11).

**Figure 12:**
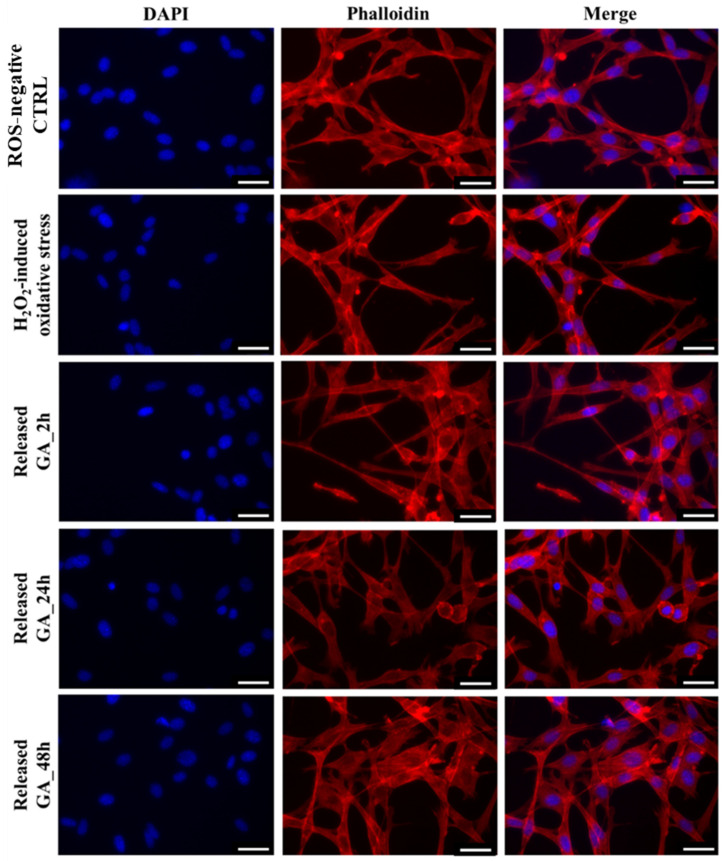
Immunofluorescence staining of NIH-3T3 cells activated with 500 µM H_2_O_2_ to induce oxidative stress (ROS-positive control) and treated with GA released from HA/D-DHP407_6%_ddH_2_O hydrogels after 2 h, 24 h and 48 h of incubation. Cells cultured in complete medium were compared as ROS-negative control. DAPI (blue) and Rhodamine Phalloidin (red) were used to stain nuclei and actin filaments, respectively. Scale bar: 25 µm.

Fluorescence microscopy immediately proved the retained capability of released GA to reduce intracellular ROS concentration, as the green fluorescence emitted by GA-treated cells was remarkably lower compared to the ROS-negative control and H_2_O_2_-activated cells. Moreover, such qualitative observations were further supported by fluorescence quantification, revealing a statistically significant reduction of ROS concentration with respect to the ROS-positive control irrespective of the considered time point. Hence, although a slight reduction of GA activity against ROS species was observed over time, in accordance with previous findings on in vitro tested scavenging effects (Figure 8), the here-developed system was effectively able to act as ROS scavenger by exerting a sustained activity up to 48 h. Lastly, to definitively prove that the very few green stained cells were ascribed to a reduced oxidative stress rather than to a reduced cell viability, the same samples were also subjected to immunofluorescence staining (Figure 12). As expected, no differences were observed compared to the ROS-negative control. Indeed, NIH-3T3 murine fibroblasts showed their characteristic spindle-like morphology irrespective of tested conditions.

## 3. Conclusions

In this work, versatile bioartificial hydrogels were engineered by successfully blending an amphiphilic poly(ether urethane) and a low molecular weight hyaluronic acid. Specifically, their temperature- and salt-induced polymeric chain interactions led to the formation of networks characterized by a double organization (i.e., supra- and macro-molecular). Moreover, such peculiarity also allowed the development of in situ gelling systems, thus opening the way to their application through mini-invasive procedures. In addition, bioartificial hydrogel suitability as protective systems for therapeutic agents was demonstrated through the encapsulation and release of gallic acid, known to show very labile stability in physiological-mimicking conditions. Released gallic acid showed remarkable scavenging activity against different ROS species (i.e., hydroxyl, superoxide and DPPH radicals) up to 48 h, thus avoiding the occurrence of premature chemical degradation phenomena during administration. Moreover, GA release kinetics from bioartificial hydrogels turned out to be suitable to exert a therapeutic effect. Indeed, each tested condition resulted to be able to reduce the concentration of intracellular ROS after the in vitro activation of NIH-3T3 murine fibroblast oxidative stress. Lastly, both the hydrogel and the therapeutic agent showed high cytocompatibility and did not induce any changes in the morphology of cells treated with their extracts up to 48 h. Therefore, the here-developed system shows promising features for application in chronic wound management, being able to preserve the activity of encapsulated scavenger molecules and release them in a proper way to broaden the therapeutic window up to 2 days of treatment.

## 4. Materials and Methods

### 4.1. Materials

Poloxamer^®^ 407 (P407, ${\bar{M}}_{n}$ 12,600 Da, 70% wt poly(ethylene oxide)), N-Boc diethanolamine, 1,6-hexamethylene diisocyanate (HDI), dibutyltin dilaurate (DBTDL), gallic acid (GA), H_2_O_2_ (30% wt.), Safranin O, pyrogallol solution, 1′-diphenyl-2-picrylhydrazyl (DPPH), 2′,7′-dichlorodihydrofluorescein diacetate (DCFH-DA), lithium bromide (LiBr), Trypsin-EDTA solution, Bovine Serum Albumin (BSA) and Dulbecco’s Phosphate Buffered solution (DPBS) were purchased from Merck (Milan, Italy). Hyaluronic acid (HA, ${\bar{M}}_{n}$ 90,000 Da) was purchased from Giusti Faravelli S.P.A. (Milan, Italy). Dulbecco’s Modified Eagle Medium (DMEM) and Calf Bovine Serum (BCS) were obtained from LGC Standards (Milan, Italy). MycoZap Plus was purchased from Lonza (Geleen, The Netherlands). Rhodamine Phalloidin and DAPI were obtained from Invitrogen (Waltham, MA, USA). All solvents were purchased from Carlo Erba Reagents (Milan, Italy) in analytical grade and used without further treatments if not specified.

### 4.2. Poly(Ether Urethane) Synthesis and Exposure of Secondary Amino Groups

The PEU exposing secondary amino groups used in this work resulted from a two-step synthesis procedure followed by an acid treatment to remove Boc-caging groups. Both procedures were conducted according to optimized protocols recently published by Laurano et al. [29]. Briefly, before the synthesis, reagents and solvent were treated to remove residual water: P407 was subjected to a thermal treatment (i.e., 8 h at 100 °C under dynamic vacuum followed by cooling to 40 °C); HDI was distilled under reduced pressure and stored in a desiccator at room temperature (RT) until use; N-Boc diethanolamine was kept in a desiccator under vacuum at RT; and 1,2-dichloroethane (DCE) was anhydrified by pouring the solvent over temperature-activated molecular sieves under a nitrogen atmosphere. Then, the P407 macrodiol was dissolved in DCE at 15% *w*/*v* concentration, equilibrated at 80 °C and added with HDI at 2:1 molar ratio and DBTDL at 0.1% wt./wt. with respect to the macrodiol to start the first step of the synthesis. After 45 min, the pre-polymer mixture was cooled down to 60 °C and N-Boc diethanolamine (5% *w*/*v* in DCE) was added at a 1:1 molar ratio with respect to P407 to start the chain extension step. The PEU synthesis was terminated after 90 min through the addition of anhydrous methanol (MeOH) to passivate isocyanate terminal functionalities and the polymer was collected through precipitation in petroleum ether at 4:1 *v*/*v* with respect to DCE reaction volume. Lastly, a purification step was carried out to remove the catalyzer and low molecular weight chains. To this purpose, the PEU (named DHP407) was dissolved in DCE at 20% *w*/*v*, precipitated in a mixture of diethyl ether/MeOH 98/2 *v*/*v* at 5:1 *v*/*v* with respect to DCE and collected by centrifugation (6000 rpm, 0 °C, 20 min).

Subsequently, the exposure of secondary amino groups along DHP407 chains was conducted through an acid treatment-mediated Boc removal [40]. A total of 10 g of PEU was first dissolved in 225 mL of chloroform (CF) under nitrogen flow (2 h, 250 rpm, RT); then, 25 mL of trifluoroacetic acid (TFA) were added and the reaction was carried on for 1 h by keeping the reaction conditions fixed. Next, the mixture was concentrated through a rotary evaporator system (Buchi Rotavapor Labortechnik AG, Flawil, Switzerland), washed twice with 100 mL of CF to remove TFA traces, dispersed in 200 mL of double demineralized water (ddH_2_O) at 4 °C overnight and dialyzed for 48 h to wash Boc groups out. Lastly, the polymer was collected by freeze-drying (Martin Christ ALPHA 2-4 LSC, Osterode Am Harz, Germany) and stored under vacuum until use.

The synthesized PEU exposing secondary amino groups will be referred to with the acronym D-DHP407.

### 4.3. Poly(Ether Urethane) Chemical Characterization

Size Exclusion Chromatography (SEC) analyses were performed through an Agilent Technologies 1200 Series (Santa Clara, CA, USA) equipped with a Refractive Index (RI) detector and two Waters Styragel columns (HR1 and HR4) equilibrated at 55 °C [43]. An amount of 2 mg of samples (P407, DHP407 and D-DHP407) was first dissolved in 1 mL of mobile phase (i.e., N,N-dimethylformamide (DMF, CHROMASOLV Plus, inhibitor-free, for HPLC, 99.9%) added with 0.1% *w*/*v* LiBr), and then the solution was filtered through a 0.45 μm syringe filter with a poly(tetrafluoroethylene) membrane. Number Average Molecular Weight (${\bar{M}}_{n}$), Weight Average Molecular Weight (${\bar{M}}_{w}$) and Polydispersity Index (D) were estimated by referring to a calibration curve based on poly(ethylene glycol) standards with a peak molecular weight in the range of 4–200 kDa.

Attenuated Total Reflectance Fourier Transform Infrared (ATR-FTIR) spectroscopy was performed using a Perkin Elmer Spectrum 100 (PerkinElmer, Waltham, MA, USA) equipped with an ATR accessory (UATR KRSS) with diamond crystal. Samples (P407, DHP407 and D-DHP407) were analyzed at RT in the range of 4000–600 cm^−1^ (32 scans, 4 cm^−1^ resolution) and the registered spectra were elaborated with the Perkin Elmer software.

Orange II Sodium Salt colorimetric assay was conducted on DHP407 (as a control condition) and D-DHP407 samples to quantify the number of exposed secondary amino groups according to the method proposed by Laurano et al. [29,40]. Briefly, 20 mg of polymer were first dissolved in 50 mL of dye solution (0.5 mM in ddH_2_O) adjusted at pH 3 with HCl 1 M. The electrostatic coupling reaction between dye molecules and –NH groups was carried on for 18 h at RT and in the dark. Next, samples were dialyzed for 8 days to remove unreacted dye molecules, freeze-dried (Martin Christ ALPHA 2-4 LSC, Osterode Am Harz, Germany) and solubilized (10 mg/mL) in ddH_2_O adjusted at pH 12 with NaOH 1 M to allow the desorbing reaction (2 h at RT and in the dark). Lastly, samples were centrifuged (6000 rpm, 15 °C, 10 min) for polymer removal and extracts were analyzed by measuring their absorbance at 485 nm through an UV/Vis spectrophotometer (PerkinElmer, Lambda 25, Waltham, MA, USA). -NH quantification was performed by referring to a calibration curve based on standards consisting of Orange molecules dissolved in the desorbing medium at concentrations ranging between 5–83 μM. The colorimetric assay was performed in triplicate and results were reported as mean ± standard deviation.

### 4.4. Bioartificial Hydrogel Preparation

The production of macromolecular hydrogels was conducted by dividing into two equal aliquots the required volume for the solubilization of the polymers (i.e., HA and D-DHP407) as they needed different temperatures to allow their complete dissolution. Then, the two aliquots were mixed to yield homogeneous solutions. In more detail, the required mass of HA was dissolved in a specific solvent (i.e., DPBS or ddH_2_O) at a concentration two-fold higher than the final one at 37 °C overnight. Similarly, D-DHP407 was solubilized in the same solvent used for HA dissolution and incubated at 3 °C overnight. Then, homogeneous HA and D-DHP407 solutions were mixed at RT and homogenized using a vortex stirrer (40 Hz, 2 min). The resulting systems were characterized by equal concentrations of HA and D-DHP407 ranging between 5% and 7.5% *w*/*v* (i.e., total polymer concentration between 10% and 15% *w*/*v*) (Table 1).

### 4.5. Qualitative Evaluation of the Thermo-Sensitivity of Macromolecular Hydrogels

The evaluation of the thermo-sensitivity of the here-designed hydrogels is extremely important to assess their suitability for in situ gelation at body temperature. The assessment of such feature was conducted by preparing the gelling solutions (1 mL) in Bijou sample containers (7 mL, polystyrene, 17 mm inner diameter, Carlo Erba Reagents, Milan, Italy) as described in Section 4.4. The specimens were first equilibrated in a water bath at 3 °C for 5 min and then incubated in a different water bath set at 37 °C. At defined time steps (from 1 to 10 min, 1 min incremental step), the samples were tilted and their physical state was qualitatively assessed: a NO-flow condition for 30 s indicated a “GEL” state. After each time step of incubation at 37 °C, the formulations were equilibrated for 5 min at 3 °C in a water bath to ensure the achievement of a complete sol state.

### 4.6. Rheological Characterization of Macromolecular Hydrogels

The evaluation of the rheological features of bioartificial gelling systems is important to determine the overall development of their network and the resulting mechanical response. In this work, a stress-controlled rheometer (MCR302, Anton Paar GmbH, Graz, Austria) was utilized. The experimental equipment was based on a 25 mm parallel plate configuration and a Peltier temperature control module. The samples with optimized polymeric concentration (as defined through qualitative thermo-sensitivity evaluation) were prepared as described in Section 4.4, equilibrated at 3 °C and poured onto the rheometer previously set at 3 °C. Then, plate distance was set at 0.8 mm, the normal force was imposed at 0 N and the test temperature was equilibrated for 15 min before all rheological analyses. Strain sweep tests were conducted at 37 °C to evaluate the responsiveness of the hydrogels in the strain range from 0.01% to 500% (frequency 1 s^−1^). Frequency sweep tests were conducted at 0.1% strain and 37 °C to evaluate the behavior of the formulated hydrogels as a function of angular frequency (within the 0.1–100 rad/s range). Finally, temperature ramp tests (range: 0–40 °C) were conducted at a temperature increase rate of 1 °C/min and constant shear rate (10 s^−1^) in order to determine the occurrence of a temperature-driven sol-to-gel transition. Analyses were performed in triplicate and results were reported as average profiles.

### 4.7. Bioartificial Hydrogel Responsiveness to Physiological-Like Fluids

Hydrogel’s capability to respond to the external environment by absorbing fluids was assessed by in vitro swelling tests, i.e., by weighing the samples in wet conditions at different time points. Specifically, HA/D-DHP407 formulations (1 mL) with the previously optimized polymeric concentration were first prepared in Bijou sample containers (7 mL, polystyrene, 17 mm inner diameter, Carlo Erba Reagents, Milan, Italy) as described in Section 4.4.; their initial weight was measured ($W_{gel\_0}$) and then they were equilibrated at 37 °C for 15 min to ensure complete temperature-induced gelation. Next, 1 mL of DPBS (37 °C) was added and the systems were incubated for defined time frames (2, 4, 6, 24, 30 and 48 h). At each time step, the supernatant was removed and the samples were weighed ($W_{gel\_f}$). The percentage of fluid absorption (%) was calculated according to Equation (1):

(1) $$Swelling\;\left(\%\right)=\frac{W_{gel\_f}-W_{gel\_0}}{W_{gel\_f}}\times 100$$

### 4.8. Preparation of Gallic Acid-Loaded Hydrogels

GA-loaded hydrogels were prepared by considering only the optimal HA/D-DHP407 system in terms of both polymeric concentration and solvent. In order to easily encapsulate GA within the hydrogels at a suitable concentration for the treatment of wound infections (i.e., 100 μg/mL) [66], a GA stock solution at 5 mg/mL concentration was initially prepared in ddH_2_O. Subsequently, 20 μL were added to gelling formulations (1 mL) prepared as described in Section 4.4 and the resulting systems were accurately mixed utilizing a vortex stirrer (40 Hz, 30 s).

### 4.9. Gallic Acid Release Study

The evaluation of GA delivery kinetics from the developed formulations was conducted through in vitro tests consisting of the direct contact of bioartificial hydrogels with a physiological-like aqueous environment (i.e., DPBS) at 37 °C. Hydrogels (1 mL) encapsulating GA at 100 μg/mL were first prepared as described in Section 4.8 and equilibrated at 37 °C for 15 min, and then 1 mL of DPBS (pH 7.4, 37 °C) was gently added to each hydrogel system. At defined time frames (i.e., 2, 4, 6, 24, 30 and 48 h) the extracts were collected and refreshed with DPBS. The quantification of GA was conducted through UV/Vis spectrophotometry (Perkin Elmer Lambda 25, Waltham, MA, USA) by measuring the absorbance at 260 nm. As a reference, a calibration curve was obtained through standard samples prepared by dissolving GA in DPBS at various concentrations ranging between 1 and 20 μg/mL. Moreover, the Korsmeyer–Peppas (Power law, Equation (2)) model was implemented as reported by Torchio et al. [44], to better evaluate the release mechanism of GA from the here-formulated hydrogels.

(2) $$\frac{M_{t}}{M_{\infty }}=Kt^{n};Log\;\left(\frac{M_{t}}{M_{\infty }}\right)=nLog\left(t\right)+Log\left(K\right)$$

where *M_t_* is the mass of released drug at a defined time step *t*, *M_∞_* is the mass of encapsulated GA and *K* is a constant of incorporation of structural modification. The analyses were conducted in triplicate and results were reported as average ± standard deviation.

### 4.10. In Vitro Assessment of ROS Scavenging Effects

The capability of released GA to exhibit ROS scavenging properties was in vitro tested on hydroxyl, superoxide and DPPH radicals. To this purpose, in vitro GA release tests were first conducted according to the method described in Section 4.9 to collect the supernatant at 2 h, 24 h and 48 h; then, such extracts were used as GA-loaded samples for the following tests. Corresponding control samples were prepared and kept at 37 °C for 2 h, 24 h and 48 h, respectively, to avoid differences ascribable to the incubation time.

Hydroxyl radical scavenging properties were tested through the Fenton reaction [67]. Briefly, 300 µL of each collected sample was first mixed with 600 µL of 2 mM FeSO_4_ solution and 500 µL of Safranin O (360 µg/mL) and equilibrated for 10 min at RT; then, 800 µL of H_2_O_2_ at 6% wt./wt. were added before incubation at 55 °C for 1 h. Control samples were prepared according to the same procedure, but extract (300 µL) and H_2_O_2_ (800 µL) were replaced with equal DPBS and ddH_2_O volumes, respectively. Lastly, sample absorbance at 517 nm was measured using an UV/Vis spectrophotometer (Perkin Elmer, Lambda 25, Waltham, MA, USA) and scavenging percentages were calculated according to Equation (3) [68].

The scavenging effects on superoxide radicals were explored through the pyrogallol assay according to a recently published study [69]. Briefly, 300 µL of each GA-containing sample was first mixed with 720 µL of Tris-HCl (50 mM, pH 8.1) and 120 µL of 3 mM pyrogallol solution; then, the reaction was carried on for 5 min at RT and in the dark and stopped by adding 240 µL of HCl 1 M. Control samples were prepared by replacing the GA-containing solution with an equal volume of DPBS. Lastly, sample absorbance was measured at 299 nm and the scavenging effect was calculated using Equation (3).

DPPH radical scavenging effects were investigated as follows: 300 µL of each GA-containing sample were first added with 1 mL of ethanol and 100 µL of DPPH solution (0.5 mM in ethanol). Then, the reaction was carried out for 60 min at RT and in the dark. Control samples were prepared by replacing the 300 µL of GA-containing extract with an equal volume of DPBS. Lastly, sample absorbance was measured at 517 nm and the scavenging effect was calculated according to the following equation:

(3) $$\mathrm{Scavenging}\;\mathrm{effect}\;(\%)=\frac{A_{control}\;-\;A_{sample}}{A_{control}}\times 100$$

All these tests were performed in quintuplicate and results were reported as mean values ± standard deviation.

### 4.11. Preliminary Assessment of Hydrogel Cytocompatibility

#### 4.11.1. Cytocompatibility of Hydrogel Extracts

CellTiter-Blue^®^ cell viability assay (Promega, Milan, Italy) was used to test the cytocompatibility of the extracts collected from the incubation of the optimized HA/D-DHP407 formulation (in terms of both polymeric concentration and solvent) with complete medium (i.e., DMEM added with MycoZap PLUS and BCS at 10% *v*/*v*) according to the ISO 10993-5 regulation [64]. To this purpose, bioartificial hydrogels (250 mg) were prepared in Bijou sample containers as described in Section 4.4 and equilibrated at 37 °C for 15 min. Then, pre-heated complete medium (1 mL per 100 mg of sample) was gently added and the systems were incubated for 2 h and 24 h at 37 °C. Extracts were collected and filtered through a 0.22 µm filter (poly(ether sulfone) membrane, Carlo Erba Reagents, Milan, Italy) under a sterile hood to provide sterility.

Meanwhile, NIH-3T3 murine fibroblasts (ATCC^®^ CRL-1658, LGC Standards, Milan, Italy) regularly cultured in complete medium at 37 °C, 5% CO_2_ in a humidified incubator, were seeded in a 96-well plate at 10,000 cells/well and cultured for 24 h to allow adherence. Before the assay, cells were tested for mycoplasma contamination (MycoAlert PLUS mycoplasma detection kit, Lonza, Galeen, The Netherlands).

Next, the complete medium was replaced with 100 µL of hydrogel extracts and the multiwell plate was again incubated for 24 h in normal culture conditions. According to the manufacturer’s instructions, cells cultured in complete medium and treated with the lysing solution provided by the kit were considered negative and positive cytotoxicity controls, respectively. Afterward, 20 µL of CellTiter-Blue^®^ solution were added to each well to allow resazurin conversion into resorufin by metabolically active cells (3 h in culture conditions). Resorufin fluorescence was finally detected through a multimode plate reader (Victor X3, Perkin Elmer, Waltham, MA, USA) at Ex/Em 535/590 nm. Lastly, cell viability was expressed as a percentage by referring to the negative cytotoxicity control (i.e., cells cultured in a complete medium). Analyses were performed in triplicate and results were reported as average values ± standard deviation.

#### 4.11.2. Cytocompatibility of Gallic Acid

A dose–response GA curve test was performed to assess the minimum therapeutic agent concentration responsible for cytotoxicity. To this purpose, NIH-3T3 murine fibroblasts were first cultured for 24 h in a 96-well plate (7500 cells/well) at 37 °C, 5% CO_2_ to allow adherence; then, the medium was replaced with GA solutions prepared by dissolving the compound in complete medium at concentrations ranging between 1.563 and 200 µg/mL and the plate was again incubated in normal culture conditions for 24 h. Afterward, the CellTiter-Blue^®^ cell viability assay (Promega, Milan, Italy) was conducted according to the manufacturer’s instructions as previously described. Cells cultured in a complete medium and treated with a lysing solution according to the kit instructions were considered negative and positive cytotoxicity controls, respectively. Before the test, the absence of mycoplasma contamination was assessed through MycoAlert PLUS mycoplasma detection kit (Lonza, Galeen, The Netherlands). Analyses were performed in triplicate and cell viability was expressed as a percentage with respect to cells cultured in complete medium.

### 4.12. Cell Protective Effect Exerted by Released GA

#### 4.12.1. Induction of an In Vitro ROS Microenvironment

The in vitro ROS microenvironment was achieved by stimulating NIH-3T3 murine fibroblasts with H_2_O_2_. To this purpose, cells were first seeded in a 96-well plate at 10,000 cells/well and cultured in normal conditions (i.e., 37 °C, 5% CO_2_) for 24 h. Then, the complete medium was replaced with 100 µL of DMEM serum-free enriched with hydrogen peroxide at different concentrations ranging between 100 µM and 1 mM to induce oxidative stress and the multiwell plate was incubated for 3 h. Afterward, the supernatant was removed, the cells were washed twice with PBS and the achievement of a ROS microenvironment was finally assessed through DCFH-DA staining. Briefly, 100 µL of DCFH-DA 10 µM were added to each well before incubation for 30 min in normal culture conditions. Afterward, the medium was removed, the cells were washed twice with DMEM serum-free and, lastly, fluorescence was measured through a multimode plate reader (Victor X3, Perkin Elmer, Waltham, MA, USA) at Ex/Em 495/529, while images were acquired through a fluorescence microscope (Leica Microsystems, Wetzlar, Germany). Cells cultured in complete medium and stained with DCFH-DA according to the same procedure were used as ROS-negative control condition.

#### 4.12.2. Measurement of Intracellular ROS under GA Scavenging Activity

To assess the scavenging activity exerted by released GA, intracellular ROS were measured after treating the cells with hydrogel extracts and inducing oxidative stress. To this purpose, GA-enriched media were first collected at 2 h, 24 h and 48 h according to the procedure previously described for the in vitro drug release test (see Section 4.9). Then, NIH-3T3 murine fibroblasts were seeded in a 96-well plate at 10,000 cells/well and cultured for 24 h in normal culture conditions to allow adherence. Afterward, the complete medium was removed and replaced with 100 µL of GA-enriched hydrogel extracts. A complete medium was used as a negative control condition. After 2 h of culture, a ROS microenvironment was induced as described in Section 4.12.1 by using 500 µM as optimized H_2_O_2_ concentration. After 2.5 h, the supernatant was removed and the cells were washed twice with PBS and stained with 100 µL of DCFH-DA 10 µM to quantify intracellular ROS as previously described (see Section 4.12.1). NIH-3T3 cultured in complete medium and NIH-3T3 added with 500 µM H_2_O_2_ without GA treatment were used as ROS-negative and ROS-positive control conditions, respectively. Analyses were performed in quintuplicate and results were reported as average values ± standard deviation. Moreover, fluorescence images of treated cells were also acquired through fluorescence microscopy (Leica Microsystems, Wetzlar, Germany).

### 4.13. Immunofluorescence Staining to Study Morphological Changes in Fibroblast Cytoskeleton

Immunofluorescence staining was conducted to assess the morphology of cells untreated and treated with GA-enriched media under oxidative stress compared to cells cultured in normal culture conditions. To this purpose, NIH-3T3 cells were first seeded at 20,000 cells/well in a 48-well plate and incubated for 24 h to allow adherence. After that, the complete medium was replaced with 300 µL of GA-enriched extracts collected at 2 h, 24 h and 48 h as described in Section 4.9 and incubated for 2 h in normal culture conditions. Then, oxidative stress was induced as described in Section 4.12.1. Cells cultured in complete medium were considered as a control condition. Afterward, cells were first fixed with paraformaldehyde (PFA, 4% *v*/*v*) for 15 min and washed twice with PBS; then, Triton was added at 0.5% for 8 min for cell membrane permeabilization. Lastly, cells were washed again with PBS and BSA was added at 1% *w*/*v* concentration before incubation for 30 min. Actin filament staining was carried out by adding 200 µL/well of Rodhamine Phalloidin solution prepared according to the manufacturer’s instructions and incubating the plate for 45 min at RT. Then, nuclei staining was performed by adding 300 µL of 300 nM DAPI solution to each well and waiting 3 min before cell washing with PBS. Lastly, images were acquired through a fluorescence microscope (Leica Microsystems, Wetzlar, Germany).

### 4.14. Statistical Analysis

Statistical analysis was performed using GraphPad Prism 8.0 (GraphPad Software version 5.03 for windows, La Jolla, CA, USA; www.graphpad.com, accessed on 1 March 2023). Two-way ANOVA analysis followed by Tukey’s multiple comparisons test was used to compare results. The statistical significance of each comparison was assessed as follows: *p* < 0.0001 = ****, 0.0001 < *p* < 0.001 = ***, 0.001 < *p* < 0.01 = **, 0.01 < *p* < 0.05 = *, *p* > 0.05 = ns.

## Figures and Tables

**Table 1:** List of acronyms used to refer to the developed HA/D-DHP407 hydrogel formulations.

	HA (% *w*/*v*)	D-DHP407 (% *w*/*v*)	Solvent
HA/D-DHP407_5%_ddH_2_O	5.0%	5.0%	ddH_2_O
HA/D-DHP407_6%_ddH_2_O	6.0%	6.0%	ddH_2_O
HA/D-DHP407_7.5%_ddH_2_O	7.5%	7.5%	ddH_2_O
HA/D-DHP407_5%_DPBS	5.0%	5.0%	DPBS
HA/D-DHP407_6%_DPBS	6.0%	6.0%	DPBS
HA/D-DHP407_7.5%_DPBS	7.5%	7.5%	DPBS

## Data Availability

The data presented in this study are available on request from the corresponding author.
